# Editorial: Beneficial and pathological roles of myeloid cells during COVID-19

**DOI:** 10.3389/fimmu.2023.1194826

**Published:** 2023-04-04

**Authors:** Lokesh Sharma, Shaon Sengupta, Thierry Roger

**Affiliations:** ^1^ Section of Pulmonary, Critical Care and Sleep Medicine, School of Medicine, Yale University, New Haven, CT, United States; ^2^ The Children’s Hospital of Philadelphia, University of Pennsylvania Perelman School of Medicine, Philadelphia, PA, United States; ^3^ Infectious Diseases Service, Department of Medicine, Lausanne University Hospital and University of Lausanne, Epalinges, Switzerland

**Keywords:** COVID - 19, neutrophils (PMNs), macrophage, SARS-CoV-2, infection - immunology

Since its emergence, coronavirus disease-2019 (COVID-19) disease has become one of the greatest challenges to human health. Extensive research efforts have been devoted to the development of novel antiviral agents and effective vaccinations against severe acute respiratory syndrome coronavirus 2 (SARS-CoV-2), the etiologic agent of COVID-19. In comparison, there are relatively few studies investigating the role of an innate immune response during COVID-19, especially that mediated by myeloid cells. Myeloid cells including monocytes, macrophages, neutrophils, and dendritic cells play a key role in host antiviral response as well as in the pathological processes that occur in infected individuals. Antiviral mechanisms mediated by myeloid cells include virus sensing and phagocytosis, production of cytokines, and activation of the adaptive immune response through antigen presentation. However, myeloid cells can also trigger hypercytokinemia and release tissue-damaging factors that contribute to the host pathology. In this Research Topic, we aimed to include studies that investigated beneficial and pathological mechanisms of myeloid cells in the pathogenesis of COVID-19.

Neutrophils, the most prominent leukocyte subpopulation in the blood, are known to have profound tissue-damaging effects that result from the release of soluble proteases and reactive oxygen species. DNA released as neutrophil extracellular traps (NETs) may have significant adverse effects participating to lung pathology and inflammation. Moreover, elevated levels of neutrophil-derived calprotectin have been associated with severe COVID-19 disease, however, its role in the pathogenesis of COVID-19 is not well understood. Loh et al. investigated the molecular mechanisms by which neutrophils sense and respond to SARS-CoV-2 to produce calprotectin, a neutrophil protein. Loh et al. described a critical role of Dok-3 in limiting calprotectin secretion by deactivating MyD88 and subsequent downstream JAK2-STAT3 signaling, revealing potential therapeutic targets in COVID-19. Another facet of the role of neutrophils is highlighted by Calvert et al. who used co-culture models to reveal that the presence of neutrophils with epithelial cells increases cytokine/chemokine response and weakens epithelial barrier function. Furthermore, neutrophils enhance viral replication and increase SARS-CoV-2 infection of the epithelium including basal stem cells. The authors confirm the deleterious role played by neutrophils using autopsy studies on the lungs of patients who died from COVID-19. Overall, these studies underline the pathological role of neutrophils during severe COVID-19.

A proportion of children with COVID-19 show signs of hyperinflammation with multi-organ involvement, defining a new syndrome called multisystem inflammatory syndrome in children (MIS-C). MIS-C has symptoms consistent with Kawasaki disease. Further exploring neutrophil biology in COVID-19, Chen et al. use whole blood single-cell RNA sequencing to compare neutrophil response in Kawasaki disease and COVID-19 disease. They demonstrate that neutrophils from these two diseases have similar transcriptomic profiles, characterized by neutrophil activation and low MHC class II expression. Confirming the role of neutrophil activation in lung pathology, Prével et al. report that high blood levels of biomarkers of neutrophil extracellular traps (NETs) are associated with disease severity. NET biomarkers are most prominently associated with acute respiratory distress syndrome (ARDS) and survival, but not pulmonary embolism. These apparently conflicting observations may be due to the limited number of patients analyzed. The authors propose that NET biomarkers be used as prognostic biomarkers in COVID-19, and that NETosis be targeted early during disease development to prevent the development of ARDS.

Beyond original research on neutrophil biology, two insightful reviews summarize specific aspects of the role of neutrophils in COVID-19. McKenna et al. provide a comprehensive review of potential mechanisms by which neutrophils contribute to lung injury and host response in SARS-CoV-2 infection. They highlight the changes in neutrophils, both in abundance and transcriptome profile, during COVID-19 disease. Further, they discuss how neutrophils may contribute to injury through the production of NETs and activation of inflammasomes. Potential approaches to target excessive neutrophil infiltration and activation in COVID-19 are proposed as possible therapeutic strategies. Along similar lines, Ventura-Santana et al. review the role of NETs in mediating viral sepsis in COVID-19. Consistent with the observations reported by Calvert et al. and Prével et al., the authors propose that direct viral infection of neutrophils exacerbates tissue damage *via* the release of NETs without aiding viral clearance.

Macrophages are one of the first responders to viral infection including by SARS-CoV-2. Atmeh et al. investigate the host-virus interaction using monocyte-derived macrophages. They report that α, β, γ, δ and o strains of SARS-CoV-2 successfully infect, but do not replicate in monocyte-derived macrophages. Further they demonstrate that infected macrophages are able to activate a γδ T cell line to secrete IFNγ and TNF. Schroeder and Beineman show that myeloid cells isolated from human blood such as basophils, dendritic cells and monocytes respond to S1, S2, and S1/S2 subunits of the spike protein (S) of SARS-CoV-2. The magnitude of the response, measured by the secretion of cytokines and chemokines, differs according to the cell type and S subunit. Monocytes respond robustly to S1 stimulation by releasing IL-1β, IL-6 and TNF, which are abundantly expressed in severe COVID-19 patients.

Finally, Trombetta et al. used multiparameter flow cytometry to investigate the frequency and phenotype of myeloid cells in patients with severe COVID-19 analyzed at admission and discharge from intensive care units (ICU). The authors describe major alterations, especially in the monocyte population. The levels of M-2 like classical monocytes are high in patients with SARS-CoV-2 viremia at ICU admission, increased at time of ICU discharge, and correlate with SARS-CoV-2 specific antibody response. In contrast, Slan^+^ non-classical monocytes are expressed at low levels in patients regardless of disease severity. These data support the idea that the resolution of acute infection and recovery from COVID-19 are related to the acquisition of an immunoregulatory phenotype by monocytic cells.

In summary, this Research Topic includes a collection of original research articles and insightful reviews supporting the role of myeloid cells in the pathogenesis of COVID-19. The key mechanisms discussed in the articles offer insights into the study of host antiviral responses. We believe that this Research Topic will stimulate interest in the role of myeloid cells in COVID-19 and for research to contribute to the development of strategies improving the management of infected patients.

## Author contributions

LS, SS, and TR have contributed to the writing of this editorial article. All authors contributed to the article and approved the submitted version.

